# Psychometric properties of morning joint stiffness duration and severity measures in patients with moderately to severely active rheumatoid arthritis

**DOI:** 10.1186/s12955-017-0813-7

**Published:** 2017-12-06

**Authors:** Elizabeth D. Bacci, Amy M. DeLozier, Chen-Yen Lin, Carol L. Gaich, Terence Rooney, Richard Hoffman, Kathleen W. Wyrwich

**Affiliations:** 1 Evidera, Inc, 1417 4th Avenue, Suite 510, Seattle, WA 98101 USA; 2 Eli Lilly and Company, Indianapolis, IN USA; 3 Evidera, Inc, Bethesda, MD USA

**Keywords:** Rheumatoid arthritis, Morning joint stiffness, Patient-reported outcome, Psychometric, Reliability, Validity, Responsiveness

## Abstract

**Background:**

To assess the measurement properties of two single-item patient-reported outcome (PRO) measures that assessed the length of time (in minutes) and severity of morning joint stiffness (MJS) experienced each day.

**Methods:**

Data from two Phase 3, randomized placebo-controlled (and active-controlled [RA-BEAM]), clinical studies assessing the safety and efficacy of baricitinib in adults with moderately to severely active rheumatoid arthritis (RA) were used to evaluate the psychometric properties of the Duration of MJS and Severity of MJS PROs.

**Results:**

Test-retest reliability of Duration of MJS and Severity of MJS was supported through large intraclass correlation coefficients among stable patients (coefficient range for both studies: 0.88 to 0.93). In support of construct validity, moderate correlations were evidenced between Duration of MJS and other related patient- and clinician-reported assessments of RA symptoms and patient functioning, whereas moderate-to-strong correlations were evidenced between these same patient- and clinician-reported assessments and Severity of MJS. Statistically significant differences between the median and mean values of Duration of MJS and Severity of MJS for differing categories of RA disease severity supported known-groups validity. Finally, large and statistically significant differences in change scores from Day 1 to Week 12 for patients defined as responders versus non-responders using the American College of Rheumatology 20 criteria supported the responsiveness of both PROs.

**Conclusion:**

Duration of MJS and Severity of MJS PROs demonstrated reliability, validity, and responsiveness in adults with moderately to severely active RA, supporting the measurement of these key symptoms in clinical trials.

**Electronic supplementary material:**

The online version of this article (doi: 10.1186/s12955-017-0813-7) contains supplementary material, which is available to authorized users.

## Background

Rheumatoid arthritis (RA) is a systemic, inflammatory, autoimmune disease. The expression and outcomes of the disease vary, ranging from mild, limited disease to severe disease that is associated with progressive joint destruction, significantly compromised health-related quality of life (HRQOL), and reduced survival. Some clinical symptoms of RA, such as morning joint stiffness (MJS), may follow a circadian pattern and the rhythm of pro-inflammatory cytokines, such as interleukin-6 (IL-6). The increase in nocturnal anti-inflammatory cortisol seen in patients with RA is generally insufficient to suppress the ongoing joint inflammation, often resulting in joint stiffness in the morning [1–3].

Prior research illustrates the importance of early morning stiffness or difficulty moving joints for patients with RA. In a study of 916 patients with recent onset of RA (disease duration ≤24 months), Westhoff et al. [4] reported that for many patients, morning stiffness impacts their HRQOL, functional capability, and ability to continue to work. These findings are supported by recent survey research conducted by Mattila et al. [5] in patients with RA (duration >6 months) who experience impairment of morning function at least 3 times per week. Of the 534 working respondents, 47% indicated morning stiffness affected their work performance, and 33% indicated it led to a late arrival at work. Of the 224 retired participants, 159 (71%) stopped working earlier than their expected retirement age, with 64% of these participants giving RA-related morning stiffness as a reason for their retirement [5]. Indeed, MJS is an important and clinically meaningful element of disease activity [6].

Patient-reported outcome (PRO) measures of symptoms like MJS are important tools to aid clinicians in treating patients with RA, facilitate doctor-patient communication to improve the quality of patient care, and contribute to better patient outcomes [7]. Despite the importance of MJS to patients with RA [4, 6], the assessment of MJS is not currently a measured component in several recommended endpoints in clinical trials, such as the American College of Rheumatology (ACR), Disease Activity Score modified to include the 28 diarthrodial joint count (DAS28), Clinical Disease Activity Index (CDAI), or Simplified Disease Activity Index (SDAI).

To address this need, two daily electronic PRO diary items were created to assess both the duration and severity of MJS from the patient’s perspective. The content validity of these two items were supported through a targeted literature review, interviews with health-care providers, and qualitative concept elicitation and cognitive debriefing interviews with patients with RA [8]. These interviews confirmed the relevance of duration and severity of MJS as symptoms of RA, as well as the appropriateness of terminology used to assess these symptoms.

This study reports the assessment of the psychometric properties (i.e., reliability, validity, and responsiveness) of two PRO items administered daily, the Duration of MJS and Severity of MJS, in patients with moderately to severely active RA who participated in two Phase 3 clinical trials for baricitinib RA-BEAM and RA-BUILD.

## Methods

### Patient population

#### RA-BEAM

RA-BEAM (*N* = 1305) was a randomized, double-blind, double-dummy, placebo- and active-controlled, parallel-arm, 52-week study designed to assess improvements in disease activity, structural preservation, and PROs including physical function, safety, and tolerability with oral baricitinib 4-mg once daily in patients with RA who had inadequate responses to methotrexate. Full details regarding the primary efficacy and safety outcomes of this study have been reported previously [9]. Briefly, patients were aged ≥18 years with active RA (≥6/68 tender and ≥6/66 swollen joints; serum high-sensitivity C-reactive protein [hsCRP] ≥6 mg/L). Comparisons were made to placebo and to adalimumab, a tumor necrosis factor (TNF)-α inhibitor and a standard-of-care biologic disease-modifying antirheumatic drug (DMARD) in this setting.

#### RA-BUILD

RA-BUILD (*N* = 684) was a randomized, double-blind, placebo-controlled, parallel-group 24-week study designed to assess improvements in disease activity, structural preservation, and PROs including physical function, safety, and, tolerability with oral baricitinib 2-mg and 4-mg once daily in patients with RA who were refractory to or intolerant of conventional synthetic DMARDs (csDMARDs). Full details regarding the primary efficacy and safety outcomes of this study have been reported previously [10]. Briefly, patients were aged ≥18 years with active rheumatoid arthritis (≥6/68 tender and ≥6/66 swollen joints; hsCRP ≥3.6 mg/L [upper limit of normal 3.0 mg/L]) and an insufficient response (despite prior therapy) or intolerance to ≥1 csDMARDs. Comparisons were made to placebo.

For both studies, visits occurred on Weeks 0, 1, 2, 4, 8, 12, 14, 16, 20, and 24, and for RA-BEAM, the visits continued to Weeks 28, 32, 40, and 52. Both studies were conducted with informed consent, under institutional review board approval, and in accordance with the Declaration of Helsinki (ClinicalTrials.gov number NCT01710358 [RA-BEAM] and NCT01721057 [RA-BUILD]).

### Instruments used in the psychometric analyses

#### Patient-reported outcomes (PROs)

##### Duration of morning joint stiffness, severity of morning joint stiffness, severity of worst tiredness, and severity of worst joint pain

Duration of MJS is a single-item PRO designed to capture information on self-reported length of time, in minutes, that a patient’s MJS lasted each day. Specifically, patients were asked: “Please indicate how long your morning joint stiffness lasted today,” and responded with the number of hours and minutes. Durations recorded as >12 h (720 min) were censored at 720 min.

Severity of MJS, Severity of Worst Tiredness, and Severity of Worst Joint Pain are all single-item PROs designed to capture the severity of MJS, worst tiredness, and worst joint pain experienced that day, respectively. Patient’s were asked to complete each at the end of their day. Specifically, for Severity of MJS, patients were asked: “Please rate the overall level of morning joint stiffness you had from the time you woke up today.” All three of these PROs are anchored at 0 and 10, where 0 represents “no joint stiffness,” “no tiredness,” or “no joint pain,” and 10 represents “joint stiffness as bad as you can imagine,” “tiredness as bad as you can imagine,” or “joint pain as bad as you can imagine,” respectively.

For RA-BEAM and RA-BUILD, all four PROs were assessed using an electronic diary on a daily basis through Week 12. The Day 1 assessment was the first assessment at the end of the patient’s day following the randomization visit (Week 0, Visit 2). The Week 1 assessment refers to the weekly average values from Days 2 to 8. Assessments at Weeks 2, 4, 8, and 12 refer to weekly average values of the 7 days prior to Weeks 2, 4, 8, and 12 visits, respectively. Recognizing that late-shift workers, individuals who work outside of the hours of 9 am until 5 pm, could not complete the electronic diary (at home) at the end of Day 1, the Day 2 assessment (if available) was used to impute missing Day 1 values so that more patients could be included in the psychometric analyses utilizing the Day 1 data.

##### Medical Outcomes Study 36-item Short Form Health Survey version 2 Acute (SF-36)

The SF-36 is a generic, 36-item PRO that measures general health status. The SF-36 includes eight domains of health status evaluated over the previous week: physical function, role limitations–physical, bodily pain, general health perceptions, vitality, social function, role limitations–emotional, and mental health. Two component scores, the Physical Component Score (PCS) and the Mental Component Score (MCS), are derived based on the 8 domain scores [11]. Domain and component scores are derived using established formulas [11], with higher scores indicating better health status or functioning. Acceptable psychometric properties of this instrument have been demonstrated elsewhere [12, 13].

##### Health Assessment Questionnaire-Disability Index (HAQ-DI)

The HAQ-DI assesses patients’ physical function or disability over the past week. The HAQ-DI contains 24 questions that query the degree of difficulty a person has in accomplishing tasks in 8 functional areas (dressing, arising, eating, walking, hygiene, reaching, gripping, and activities). Responses in each functional area are scored from 0, indicating “no difficulty,” to 3, indicating “inability to perform a task” in that area. The HAQ-DI total score, ranging from 0 to 3 (higher values indicate worse functioning), is obtained by summing the highest score within each functional area and dividing by the number of functional areas answered [14]. The reliability and validity of this instrument have been documented previously [15].

##### Quick Inventory of Depressive Symptomatology Self-Rated-16 (QIDS-SR_16_)

The QIDS-SR_16_ is a 16-item PRO intended to assess the existence and severity of symptoms of depression as listed in the American Psychiatric Association’s Diagnostic and Statistical Manual of Mental Disorders, 4th Edition (DSM-IV) [16]. Patients were asked to consider each statement as it relates to the way they have felt for the past 7 days. There is a unique 4-point ordinal scale for each item, with scores ranging from 0 to 3 reflecting increasing depressive symptoms as the item score increases. The instrument measures 9 core symptom domains that are used to define a depressive episode: sad mood; concentration; self criticism; suicidal ideation; interest; energy/fatigue; sleep disturbance; decrease or increase in appetite or weight; and psychomotor agitation or retardation. The QIDS-SR_16_ total score is derived as the sum of the scores across the 9 scale domains. The psychometric properties, including reliability, validity, and sensitivity to change of this instrument have been demonstrated elsewhere [17].

##### Patient’s assessment of pain

Patient’s current pain was assessed at each study visit with the use of a 0- to 100-mm visual analogue scale (VAS), with higher scores indicating more severe pain.

##### Patient's Global Assessment of Disease Activity (PtGA)

The PtGA, assessed as their current disease activity, was assessed at each study visit and is recorded on a 0- to 100-mm VAS, with higher scores indicating more active RA.

#### Clinician-reported assessments

##### Physician's Global Assessment of Disease Activity (PhGA)

The PhGA was assessed at each study visit and is recorded on a 0-to 100-mm VAS, with higher scores indicating or more active RA.

#### Clinical signs and symptoms measures

##### American College of Rheumatology 20 (ACR20)

An ACR20 response (i.e., a binary variable indicating achieving or not achieving a response) was measured at each study visit and is defined as at least a 20% improvement from baseline in both tender joint count (TJC) (0 to 68) and swollen joint count (SJC) (0 to 66), and in at least 3 of the following 5 assessments: patient’s assessment of pain, PtGA, PhGA, HAQ-DI, and high-sensitivity C-reactive protein.

##### Clinical Disease Activity Index (CDAI)

The CDAI is a tool for measurement of disease activity in RA that integrates measures of physical examination, patient self-assessment, and evaluator assessment [18]. The CDAI was assessed at each study visit and is calculated by adding together scores from the following assessments: number of swollen joints (0 to 28), number of tender joints (0 to 28), PtGA on a VAS (0 to 10 cm), PhGA on a VAS (0 to 10 cm). Total scores are calculated using established formulas [18]. Thresholds have been established for the CDAI (remission: 0.0 to ≤2.8; low disease activity: >2.8 to ≤10; moderate disease activity: >10 to ≤22; high disease activity: >22 to ≤76) [19].

##### Disease Activity Score (28 joints) (DAS28)

The DAS28 is a composite score that is based on a 28-joint count (both TJC 0 to 28 and SJC 0 to 28), hsCRP or erythrocyte sedimentation rate (ESR), and PtGA and was measured at each study visit. Total scores are calculated using established formulas [20]. Patients can be categorized into 4 groups (remission: <2.6; low disease activity: ≥2.6 to ≤3.2; moderate disease activity: >3.2 to ≤5.1; high disease activity: >5.1).

### Statistical analyses

The distribution of scores for the Duration of MJS and Severity of MJS PROs was assessed using descriptive statistics at Day 1, including mean (SD), median, range, and ceiling/floor effects.

#### Reliability (test-retest)

Test-retest reliability was assessed in stable patients during the interval between Week 1 and 2 and again between Week 4 and 8. Stable patients were defined as those with ≤5 point difference [21] on the 0 to 100 PtGA between each assessment period. Intraclass correlation coefficients (ICCs) were calculated between the initial (Week 1 or 4) and retest (Week 2 or 8) scores to evaluate test-retest reliability. An ICC of ≥0.70 was considered good agreement [22].

#### Convergent and discriminant validity (construct validity)

Construct validity was assessed by Pearson correlations at Day 1 and Week 12 between the scores of Duration of MJS and Severity of MJS, and the scores of other clinical/PRO endpoints: Severity of Worst Tiredness, Severity of Worst Joint Pain, SF-36 domain and component scores, HAQ-DI, QIDS-SR_16_, patient’s assessment of pain, PtGA, TJC28, SJC28, PhGA, and hsCRP. Cohen’s conventions were used to interpret the absolute value of the correlation results, where a correlation >0.5 is large, 0.3 to 0.5 is moderate, 0.1 to <0.3 is small, and <0.1 is insubstantial [23].

It was hypothesized that moderate or large correlations supporting convergent validity would be demonstrated between Duration of MJS and Severity of MJS and the PRO instruments measuring concepts related to RA symptoms (SF-36 PCS, SF-36 Bodily Pain), their impact on functioning (SF-36 Social Functioning, SF-36 Vitality, SF-36 Physical Functioning, HAQ-DI), and clinician-reported/laboratory assessments of disease activity (TJC28, SJC28, PhGA, and hsCRP). Discriminant validity was assessed by Pearson correlations at Day 1 and at Week 12 between Duration of MJS, Severity of MJS, and PROs measuring distally related concepts (SF-36 MCS, SF-36 Role Emotional, QIDS-SR_16_) where small correlations were hypothesized.

#### Known-groups validity

Known-groups validity was evaluated using the Kruskal-Wallis test to distinguish median Duration of MJS and an analysis of variance (ANOVA) model to distinguish mean Severity of MJS between subgroups defined by the DAS28-ESR thresholds (<2.6; ≥2.6 and ≤3.2; >3.2 and ≤5.1; >5.1) measured at Day 1 and Week 4, and CDAI (0.0 to ≤2.8; >2.8 to ≤10; >10 to ≤22; and >22 to ≤76) measured at Day 1 and Week 4. The Scheffé adjustment was used for multiple comparisons. Subgroups were combined in instances of small sample sizes (i.e., <5% of the total sample size for the subgroup).

#### Responsiveness

Due to anticipated skewness of Duration of MJS PRO, responsiveness was evaluated using a nonparametric randomization-based analysis of covariance (ANCOVA) methodology [24] to assess significant differences in median change in Duration of MJS from Day 1 to Week 12 between ACR20 responders and nonresponders at Week 12, controlling for Duration of MJS at Day 1. Responsiveness was evaluated using an ANCOVA methodology to assess significant differences in mean change in Severity of MJS. Responsiveness was also assessed using disease activity as measured by DAS28-hsCRP at Week 12, using the following subgroups: DAS28-hsCRP <2.6, DAS28-hsCRP ≥2.6 and DAS28-hsCRP ≤3.2, and DAS28-hsCRP >3.2. An overall statistically significant difference (*p* < 0.05) with statistically significant subgroup comparisons was hypothesized.

SAS® statistical software Version 9,4 (SAS Institute Inc., Cary, NC, USA) was used to conduct all analyses.

## Results

Baseline demographics for the total modified intent-to-treat population, patients with Day 1 diary scores, and patients with Week 12 diary scores are provided in Table 1. Baseline and Week 12 scores for Duration of MJS PRO, Severity of MJS PRO, and other assessments are presented in Table 2, while score distributions for Duration of MJS PRO and Severity of MJS PRO are found in Additional file 1: Table S1. As can be seen in Tables 1 and 2, there was a large amount of missing data at the Day 1 assessment period. This missing data was due to multiple reasons as shown in Additional file 1: Table S2, such as the diary device alarm not sounding until the following day or the diary device being given to the patient after Day 1. Sensitivity analyses with the imputation for missing data at Day 1 (*n* = 1041 for RA-BEAM and *n* = 563 for RA-BUILD, respectively) demonstrated similar comparisons to the results presented in this document.

**Table 1:** Patient Demographic and Disease Characteristics for Patients with Electronic Diary Assessments at Day 1 and Patients with Week 12 Electronic Diary Assessments (mITT Population) for RA-BEAM and RA-BUILD

Characteristics	RA-BEAM			RA-BUILD		
	Total mITT Population(*n* = 1305)	Patients with Day 1 Diary Scores(*n* = 537)	Patients with Week 12 Diary Scores^a^(*n* = 1281)	Total mITT Population(*n* = 684)	Patients with Day 1 Diary Scores(*n* = 312)	Patients with Week 12 Diary Scores^a^(*n* = 666)
Age (years)						
Mean (SD)	53.3 (12.1)	53.2 (12.3)	53.2 (12.0)	51.8 (12.3)	51.5 (12.3)	51.8 (12.3)
Gender, n (%)						
Female	1009 (77.3%)	414 (77.1%)	989 (77.2%)	560 (81.9%)	262 (84.0%)	546 (82.0%)
Race, n (%)						
White	818 (62.7%)	422 (78.6%)	801 (62.5%)	457 (66.8%)	254 (81.4%)	444 (66.7%)
Black or African American	10 (0.8%)	5 (0.9%)	9 (0.7%)	28 (4.1%)	16 (5.1%)	27 (4.1%)
Asian	392 (30.0%)	45 (8.4%)	388 (30.3%)	180 (26.3%)	31 (9.9%)	176 (26.4%)
American Indian or Alaska Native	63 (4.8%)	49 (9.1%)	61 (4.8%)	14 (2.0%)	8 (2.6%)	14 (2.1%)
Native Hawaiian or Other Pacific Islander	0 (0.0%)	0 (0.0%)	0 (0.0%)	1 (0.1%)	0 (0.0%)	1 (0.1%)
Multiple	21 (1.6%)	16 (3.0%)	21 (1.6%)	3 (0.4%)	2 (0.6%)	3 (0.5%)
Missing	1 (0.1%)	0 (0.0%)	1 (0.1%)	1 (0.1%)	1 (0.3%)	1 (0.2%)
Years from RA diagnosis						
Mean (SD) [min, max]	8.7 (8.2) [0–56]	8.7 (8.0) [0–40]	8.6 (8.1) [0–56]	6.3 (7.3) [0–53]	6.2 (6.5) [0–41]	6.4 (7.4) [0–53]

Abbreviations: *max* maximum, *min* minimum, *mITT* modified intent-to-treat, *n* number of patients in the specified category, *RA* rheumatoid arthritis, *SD* standard deviation

^a^Daily average of 7 days preceding visit that contained at least 4 measurements

**Table 2:** Instrument Scores at Day 1 and Week 12 for RA-BEAM and RA-BUILD

Instrument	RA-BEAM^a^		RA-BUILD^b^	
	Day 1	Week 12	Day 1	Week 12
Duration of MJS, Mean (SD)	152.8 (180.8)	96.7 (144.7)	160.7 (174.8)	103.5 (147.5)
Severity of MJS, Mean (SD)	5.8 (2.2)	3.5 (2.4)	5.7 (2.1)	3.7 (2.4)
Severity of Worst Tiredness, Mean (SD)	5.8 (2.1)	4.0 (2.3)	6.0 (2.1)	4.1 (2.4)
Severity of Worst Joint Pain, Mean (SD)	6.1 (2.0)	4.0 (2.3)	6.0 (2.1)	4.2 (2.4)
Patient’s Assessment of Pain, Mean (SD)	60.8 (22.3)	34.9 (24.1)	58.0 (22.1)	36.5 (24.9)
Patient’s Global Assessment of Disease Activity, Mean (SD)	62.4 (21.8)	36.3 (23.5)	60.7 (21.1)	37.7 (24.0)
SF-36				
Role Physical Domain, Mean (SD)	35.5 (10.4)	41.8 (10.2)	35.3 (9.2)	40.7 (9.6)
Bodily Pain Domain, Mean (SD)	34.7 (7.8)	42.5 (8.7)	34.9 (7.6)	41.9 (9.0)
General Health Domain, Mean (SD)	36.8 (8.5)	41.6 (8.9)	36.8 (8.3)	42.0 (9.4)
Social Functioning Domain, Mean (SD)	40.8 (11.6)	45.4 (10.5)	40.2 (11.2)	44.7 (10.3)
Vitality Domain, Mean (SD)	43.7 (10.0)	49.4 (9.8)	41.9 (10.1)	48.4 (10.0)
Role Emotional Domain, Mean (SD)	41.1 (12.6)	45.5 (10.9)	40.9 (12.3)	44.8 (11.2)
Mental Health Domain, Mean (SD)	43.0 (11.3)	47.1 (10.7)	42.9 (11.6)	47.1 (11.3)
Physical Functioning Domain, Mean (SD)	32.2 (10.4)	38.7 (10.9)	32.2 (10.2)	38.2 (10.9)
Mental Component Score, Mean (SD)	46.4 (11.8)	49.8 (10.8)	45.7 (11.8)	49.3 (11.1)
Physical Component Score, Mean (SD)	32.3 (8.6)	39.4 (9.3)	32.3 (8.3)	38.8 (9.4)
QIDS-SR_16_ Total Score, Mean (SD)	8.0 (5.0)	5.9 (4.2)	8.4 (4.9)	6.2 (4.3)
HAQ-DI Total Score, Mean (SD)	1.56 (0.68)	1.03 (0.70)	1.52 (0.61)	1.03 (0.66)
PhGA, Mean (SD)	65.0 (16.9)	31.0 (21.7)	63.5 (17.4)	33.9 (22.4)
CDAI, Mean (SD)	37.83 (12.55)	18.68 (13.54)	36.05 (12.12)	18.04 (13.15)
DAS28-ESR, Mean (SD)	6.43 (0.97)	4.62 (1.46)	6.22 (0.97)	4.50 (1.37)
DAS28-hsCRP, Mean (SD)	5.73 (0.94)	3.93 (1.39)	5.55 (0.91)	3.84 (1.35)

Abbreviations: *CDAI* Clinical Disease Activity Index, *DAS28* Disease Activity Score modified to include the 28 diarthrodial joint count, *ESR* erythrocyte sedimentation rate, *HAQ-DI* Health Assessment Questionnaire-Disability Index, *hsCRP* high-sensitivity C-reactive protein, *n* number of patients, *MJS* morning joint stiffness, *PhGA* Physician’s Global Assessment of Disease Activity, *QIDS-SR*_*16*_ Quick Inventory of Depressive Symptomatology Self-Rated-16, *SD* standard deviation, *SF-36,* 36-Item Short Form Health Survey version 2 with Acute recall

^a^Daily diary responses were *n* = 537(Day 1) and *n* = 1281 (Week 12); nondiary assessments had approximately *n* = 1301 (Day 1) and *n* = 1250 (Week 12)

^b^Daily diary responses were *n* = 310 (Day 1) and *n* = 666 (Week 12); nondairy assessments had approximately *n* = 680 (Day 1) and *n* = 644 (Week 12)

### Reliability (test-retest)

For RA-BEAM and RA-BUILD, the ICCs for weekly mean Duration of MJS and Severity of MJS ranged from 0.88 to 0.91 from Week 1 to 2 (RA-BEAM *n* = 412; RA-BUILD *n* = 185) and from 0.90 to 0.93 from Week 4 to Week 8 (RA-BEAM *n* = 417; RA-BUILD *n* = 215). These values provide evidence for substantial agreement between assessment periods among stable patients.

### Convergent and discriminant validity

Results supporting convergent validity of Duration of MJS and Severity of MJS are found in Table 3. For Duration of MJS, small-to-moderate associations were found between Duration of MJS and other assessments of RA symptoms, assessments of function, and clinician-reported assessments at Day 1 and Week 12. At Day 1 and Week 12, respectively, Duration of MJS was most strongly associated with Severity of MJS (RA-BEAM *r =* 0.36 and 0.45; RA-BUILD *r =* 0.35 and 0.52) and Severity of Worst Joint Pain (RA-BEAM *r =* 0.29 and 0.41; RA-BUILD *r =* 0.30 and 0.47), followed by the patient’s assessment of pain (RA-BEAM *r =* 0.24 and 0.38; RA-BUILD *r =* 0.23 and 0.39) and PtGA (RA-BEAM *r =* 0.19 and 0.36; RA_BUILD *r =* 0.28 and 0.37).

**Table 3:** Pearson Correlations between Duration of MJS, Severity of MJS, Severity of Worst Tiredness, and Other Instruments in RA-BEAM and RA-BUILD

Instruments	Duration of MJS				Severity of MJS			
	RA-BEAM		RA-BUILD		RA-BEAM		RA-BUILD	
	Number	*r*	Number	*r*	Number	*r*	Number	*r*
Day 1								
Duration of MJS	–	–	–	–	537	0.36***	311	0.35***
Severity of MJS	537	0.36***	311	0.35***	–	–	–	–
Severity of Worst Tiredness	537	0.21***	311	0.09	537	0.66***	311	0.52***
Severity of Worst Joint Pain	537	0.29***	310	0.30***	537	0.79***	310	0.75***
SF-36								
Role Physical Domain	535	−0.24***	311	−0.23***	535	−0.43***	312	−0.40***
Bodily Pain Domain	535	−0.30***	311	−0.29***	535	−0.65***	312	−0.51***
General Health Domain	535	−0.09*	311	−0.18**	535	−0.30***	312	−0.25***
Social Functioning Domain	535	−0.16***	311	−0.24***	535	−0.38***	312	−0.41***
Vitality Domain	535	−0.21***	311	−0.18**	535	−0.42***	312	−0.32***
Role Emotional Domain	535	−0.07	311	−0.12*	535	−0.27***	312	−0.25***
Mental Health Domain	535	−0.10*	311	−0.20***	535	−0.31***	312	−0.27***
Physical Functioning Domain	535	−0.25***	311	−0.28***	535	−0.51***	312	−0.47***
Mental Component Score	535	−0.07	311	−0.15**	535	−0.26***	312	−0.25***
Physical Component Score	535	−0.29***	311	−0.27***	535	−0.54***	312	−0.44***
HAQ-DI Total Score	535	0.22***	311	0.26***	535	0.53***	312	0.46***
QIDS-SR_16_ Total Score	535	0.15***	311	0.26***	535	0.34***	312	0.28***
Patient’s Assessment of Pain	535	0.24***	311	0.23***	535	0.59***	312	0.54***
PtGA	535	0.19***	311	0.28***	535	0.59***	312	0.53***
Tender Joint Count 28	537	0.09*	311	0.15**	537	0.26***	312	0.43***
Swollen Joint Count 28	537	0.06	311	0.13*	537	0.21***	312	0.24***
PhGA	535	0.19***	304	0.19***	535	0.32***	305	0.33***
hsCRP	535	0.20***	311	0.27***	535	0.48***	312	0.53***
Week 12^a^								
Duration of MJS	–	–	–	–	1281	0.45***	666	0.52***
Severity of MJS	1281	0.45***	666	0.52***	–	–	–	–
Severity of Worst Tiredness	1281	0.32***	666	0.38***	1281	0.79***	666	0.77***
Severity of Worst Joint Pain	1281	0.41***	666	0.47***	1281	0.89***	666	0.89***
SF-36								
Role Physical Domain	1233	−0.23***	632	−0.29***	1233	−0.47***	632	−0.45***
Bodily Pain Domain	1233	−0.31***	632	−0.36***	1233	−0.59***	632	−0.55***
General Health Domain	1233	−0.15***	632	−0.19***	1233	−0.36***	632	−0.30***
Social Functioning Domain	1233	−0.18***	632	−0.30***	1233	−0.43***	632	−0.46***
Vitality Domain	1233	−0.19***	632	−0.29***	1233	−0.41***	632	−0.39***
Role Emotional Domain	1233	−0.18***	632	−0.23***	1233	−0.39***	632	−0.33***
Mental Health Domain	1233	−0.16***	632	−0.21***	1233	−0.33***	632	−0.30***
Physical Functioning Domain	1233	−0.22***	632	−0.32***	1233	−0.49***	632	−0.46***
Mental Component Score	1233	−0.14***	632	−0.21***	1233	−0.31***	632	−0.29***
Physical Component Score	1233	−0.25***	632	−0.31***	1233	−0.52***	632	−0.48***
HAQ-DI Total Score	1233	0.23***	632	0.31***	1233	0.55***	632	0.51***
QIDS-SR_16_ Total Score	1231	0.20***	632	0.24***	1231	0.34***	632	0.31***
Patient’s Assessment of Pain	1233	0.38***	632	0.39***	1233	0.78***	632	0.72***
PtGA	1233	0.36***	632	0.37***	1233	0.74***	632	0.67***
Tender Joint Count 28	1231	0.16***	630	0.31***	1231	0.39***	630	0.44***
Swollen Joint Count 28	1231	0.16***	630	0.22***	1231	0.28***	630	0.31***
PhGA	1224	0.21***	627	0.29***	1224	0.41***	627	0.44***
hsCRP	1220	0.26***	623	0.37***	1220	0.57***	623	0.57***

Abbreviations: *HAQ-DI* Health Assessment Questionnaire-Disability Index, *hsCRP* high-sensitivity C-reactive protein, *MJS* morning joint stiffness, *n* number of patients, *PtGA* Patient’s Global Assessment of Disease Activity, *PhGA* Physician’s Global Assessment of Disease Activity, *QIDS-SR*_*16*_ Quick Inventory of Depressive Symptomatology Self-Rated-16, *SF-36* 36-Item Short Form Health Survey version 2 with Acute recall

Pearson correlation coefficients with significance using *p*-values denoted as: * ≤ 0.05, ** ≤ 0.01, *** ≤ 0.001

^a^Daily average of 7 days preceding visit that contained at least 4 measurements [29]

For Severity of MJS, moderate-to-large associations were found between Severity of MJS and other assessments of RA symptoms, assessments of function, and clinician-reported assessments at Day 1 and Week 12. At Day 1 and Week 12, respectively, Severity of MJS was most strongly associated with Severity of Worst Joint Pain (RA-BEAM *r =* 0.79 and 0.89; RA-BUILD *r =* 0.75 and 0.89), patient’s assessment of pain (RA-BEAM *r =* 0.59 and 0.78; RA-BUILD *r =* 0.54 and 0.72), and Severity of Worst Tiredness (RA-BEAM *r =* 0.66 and 0.79; RA-BUILD *r =* 0.52 and 0.77).

In contrast, smaller correlations between Duration and Severity of MJS with SF-36 MCS, SF-36 Role Emotional, and the QIDS-SR_16_ at Day 1 (*r* = −0.07 to 0.34) and Week 12 (*r* = −0.39 to 0.34) were observed, indicating that the SF-36 and QIDS-SR_16_ assessments measure more distally related constructs.

These findings demonstrate adequate convergent and discriminant validity, as well as the uniqueness of Duration of MJS and Severity of MJS compared to other PROs and clinical measures of RA in patients with moderately to severely active RA.

### Known-groups validity

Due to small sample sizes in the lower DAS28-ESR subgroups at Day 1 (i.e., <5% of the sample in each score category) in RA-BEAM and RA-BUILD, patients were categorized into only 2 subgroups: ≤5.1 and >5.1 (Table 4). At Day 1 in both studies, patients with higher DAS28-ESR scores reported a significantly longer Duration of MJS measured in minutes (RA-BEAM: *p* = 0.028; RA-BUILD: *p* = 0.033) and significantly greater Severity of MJS (RA-BEAM: *p* = 0.001; RA-BUILD: *p* = 0.001) than those patients with lower DAS28-ESR scores. Similar findings were evident for both studies at Week 4.

**Table 4:** Known-Groups Validity of Duration of MJS and Severity of MJS Using DAS28-ESR Subgroups at Day 1 and Week 4, and CDAI Subgroups at Day 1

PRO/Study	DAS28-ESR Category		*p*-value, ES
	≤5.1 N, Mean (SD), Median	>5.1 N, Mean (SD), Median	
Duration of MJS Day 1			
RA-BEAM	39, 120.5 (190.8), 60.0	496, 155.3 (180.1), 90.0	0.028^a^, 0.19
RA-BUILD	36, 132.8 (191.5), 60.0	273, 164.9 (173.1), 120	0.033^a^, 0.18
Duration of MJS Week 4			
RA-BEAM	619, 76.1 (108.6), 37.3	587, 133.6 (153.5), 73.6	0.001^a^, 0.43
RA-BUILD	327, 77.6 (112.0), 40.0	286, 151.1 (158.3), 94.6	0.001^a^, 0.54
Severity of MJS at Day 1			
RA-BEAM	39, 4.4 (2.7)	496, 5.9 (2.1)	0.001^b^, 0.70
RA-BUILD	36, 4.0, (1.7)	274, 5.9 (2.0)	0.001^b^, 0.97
Severity of MJS at Week 4			
RA-BEAM	619, 3.0 (1.9)	587, 5.1 (2.1)	0.001^b^, 1.05
RA-BUILD	327, 3.1 (1.9)	286, 5.3 (2.0)	0.001^b^, 1.13
	CDAI Category		*p*-value, ES
	0.0 to ≤22.0 N, Mean (SD), Median	>22.0 to ≤76.0 N, Mean (SD), Median	
Duration of MJS Day 1			
RA-BEAM	36, 144.1 (198.3), 90.0	497, 153.9 (180.0), 90.0	0.305^a^, 0.05
RA-BUILD	26, 111.9 (162.7), 60.0	278, 162.3 (170.6), 120	0.016^a^, 0.30
Severity of MJS at Day 1			
RA-BEAM	36, 4.7 (2.4)	497, 5.9 (2.1)	0.001^b^, 0.57
RA-BUILD	26, 3.7 (1.6)	278, 5.9 (2.1)	0.001^b^, 1.07

Abbreviations: *CDAI* Clinical Disease Activity Index, *DAS28* Disease Activity Score modified to include the 28 diarthrodial joint count, *ESR* erythrocyte sedimentation rate, *MJS* morning joint stiffness, *SD* standard deviation, *ES* effect size

^a^ *p*-value based on a comparison of median values using the Kruskal-Wallis test

^b^ *p*-value based on a comparison of mean values using ANOVA

Similar to the approach taken for the DAS28-ESR analyses, due to small sample sizes in the lower CDAI subgroups, patients were categorized into 2 subgroups based on the CDAI at Day 1 (0.0 to ≤22.0 and >22.0 to ≤76.0) and 3 subgroups at Week 4 (0.0 to ≤10.0, >10.0 to ≤22.0, and >22.0 to ≤76.0). At Day 1, patients with higher CDAI scores experienced a significantly longer Duration of MJS in RA-BUILD (median = 60.0 vs. 120.0, *p* = 0.016) and greater Severity of MJS in both RA-BEAM and RA-BUILD (RA-BEAM: mean = 4.7 vs. 5.9, *p* = 0.001; RA-BUILD: 3.7 vs. 5.9, *p* = 0.001) than those patients with a lower CDAI score (Table 4). However, at Week 4, the Duration of MJS was significantly longer and the Severity of MJS was significantly greater in patients with higher CDAI scores than in patients with low CDAI scores in both studies (all *p* = 0.001) (Table 5). All values increased linearly with higher CDAI scores, where all post-hoc comparisons using Scheffé adjustment were statistically significant (*p* = 0.001), except for the comparison of median Duration of MJS values between CDAI categories of 0.0 to ≤10.0 versus >10.0 to ≤22.0 in RA-BUILD. These findings indicate that the Duration of MJS and Severity of MJS PROs are able to distinguish between known groups based on disease severity.

**Table 5:** Known-Groups Validity of Duration of MJS and Severity of MJS Using CDAI Subgroups at Week 4

PRO/Study	CDAI Category			*P*-value	Comparisons, ES^c^
	0.0 to ≤10.0 N, Mean (SD), Median	>10.0 to ≤22.0 N, Mean (SD), Median	>22.0 to ≤76.0 N, Mean (SD), Median		
Duration of MJS Week at Week 4					
RA-BEAM	220, 58.3 (95.0), 20.4	426, 90.1 (121.8), 47.1	567, 133.0 (152.8), 75.7	0.001^a^	a: 0.001, 0.28; b: 0.001, 0.54; c: 0.001, 0.31
RA-BUILD	134, 70.5 (107.1), 31.4	209, 88.1 (123.3), 45.7	275, 152.9 (158.9), 95.0	0.001^a^	a: 0.146, 0.15; b: 0.001, 0.57; c: 0.001, 0.45
Severity of MJS at Week 4					
RA-BEAM	220, 2.3 (1.7)	426, 3.6 (2.0)	567, 5.0 (2.1)	0.001^b^	a: 0.001, 0.68; b: 0.001, 1.35; c: 0.001, 0.68
RA-BUILD	134, 2.5 (1.8)	209, 3.6 (1.8)	275, 5.4 (2.0)	0.001^b^	a: 0.001, 0.61; b: 0.001, 1.50; c: 0.001, 0.94

Abbreviations: *CDAI* Clinical Disease Activity Index, *MJS* morning joint stiffness, *SD* standard deviation, *ES* effect size

^a^ *p*-value based on a comparison of median values using the Kruskal-Wallis test

^b^ *p*-value based on a comparison of mean values using analysis of variance

^c^Note for multiple comparison using Scheffé adjustment: a: 0.0 to ≤10.0 versus >10.0 to ≤22.0; b: 0.0 to ≤10.0 versus >22.0 to ≤76.0; c: >10.0 to ≤22.0 versus >22.0 to ≤76.0. Effect size estimates were calculated on the unadjusted group scores

### Responsiveness

As hypothesized, the responsiveness of the Duration of MJS and Severity of MJS PROs was supported by large and statistically significant differences in median and mean change in Duration of MJS and Severity of MJS, respectively, from Day 1 to Week 12 between patients defined as responders or nonresponders on the basis of the ACR20 at Week 12 (Table 6). For example, for Duration of MJS in RA-BEAM, median change from Day 1 to Week 12 was −35.1 for responders in comparison to −7.0 for nonresponders (*p* = 0.001). Similarly, for Severity of MJS in RA-BEAM, mean change was −3.2 compared to −1.1 for responders and nonresponders, respectively (*p* = 0.001).

**Table 6:** Change from Day 1 to Week 12 among ACR20 and DAS28-hsCRP Groups

PRO/Study/Statistics	ACR20 Category at Week 12		*P*-value, ES	DAS28-hsCRP Groups at Week 12^b^			Comparisons, ES^c^
	Responder^a^	Nonresponder^a^		DAS28-hsCRP <2.6	2.6 ≤ DAS28-hsCRP <3.2	DAS28-hsCRP ≥3.2	
Duration of MJS							
RA-BEAM							
N, Mean (SD)	326, −84.2 (167.5)	211, −17.2 (166.4)		88, −92.4 (185.0)	79, −84.0 (170.1)	368, −44.2 (165.4)	
Median ^d^	−35.1	−7.0	0.001, −0.40	−55.0	−30.0	−12.6	a: 0.809, −0.05
							b: 0.001, −0.28
							c: 0.001, −0.24
RA-BUILD							
N, Mean (SD)	174, −70.1 (139.3)	137, −19.6 (196.8)		61, −87.9 (133.7)	33, −65.7 (96.7)	214, −34.1 (185.0)	
Median ^d^	−42.9	−9.1	0.001, −0.30	−50.7	−40.0	−26.9	a: 0.221, −0.18
							b: 0.001, −0.31
							c: 0.022, −0.18
Severity of MJS							
RA-BEAM							
N, Mean (SD)	326, −3.2 (2.3)	211, −1.1 (2.2)	0.001, −0.90	88, −3.7 (2.3)	79, −3.1 (2.3)	368, −1.9 (2.4)	a: 0.083, −0.25
							b: 0.001, −0.76
							c: 0.001, −0.52
RA-BUILD							
N, Mean (SD)	175, −2.9 (2.3)	137, −1.0 (2.1)	0.001, −0.85	61, −3.1 (2.5)	33, −2.6 (2.6)	215, −1.8 (2.2)	a: 0.264, −0.21
							b: 0.001, −0.59
							c: 0.002, −0.35

Abbreviations: *ACR20* 20% improvement in American College of Rheumatology criteria, *DAS28* Disease Activity Score modified to include the 28 diarthrodial joint count, *hsCRP* high-sensitivity C-reactive protein, *MJS* morning joint stiffness, *SD* standard deviation, *ES* effect size

^a^Responder: Achievement of ACR20 criteria at Week 12; Nonresponder: Failure to achieve ACR20 criteria at Week 12

^b^Missing DAS28-hsCRP at Week 12 was imputed using modified baseline observation carried forward

^c^Note for multiple comparison: a: <2.6 vs. ≥2.6 and <3.2; b: <2.6 vs. ≥3.2; c: ≥2.6 and <3.2 vs. ≥3.2

^d^Daily average of 7 days preceding visit that contained at least 4 measurements

Comparable findings were seen when using DAS28-hsCRP as an anchor, as pairwise comparisons assessing significant differences in median and mean change between DAS28-hsCRP groups of <2.6 versus ≥3.2 (*p* = 0.001 for all comparisons), and ≥2.6 and <3.2 versus ≥3.2 were statistically significant (*p* < 0.01 for all comparisons) (Table 6). However, the change scores comparisons between <2.6 versus ≥2.6 and <3.2 were not statistically significant for Duration of MJS (RA-BEAM *p* = 0.809; RA-BUILD *p* = 0.221) or Severity of MJS (RA-BEAM *p* = 0.083; RA-BUILD *p* = 0.264).

## Discussion

The psychometric properties, including reliability, validity, and responsiveness, of Duration of MJS and Severity of MJS PROs were supported by the results from RA-BEAM and RA-BUILD. Specifically, test-retest reliability analyses revealed high levels of agreement in Duration of MJS and Severity of MJS values among patients considered stable across two assessment periods. In support of construct validity, a priori hypotheses of the relationships between Duration of MJS and Severity of MJS with other related PROs and clinical outcome measures were supported at Day 1 and Week 12 assessment periods. Known-groups validity was similarly supported as median and mean values of Duration of MJS and Severity of MJS were significantly different based on known-groups using the DAS28-ESR and CDAI. Finally, both measures were responsive to change from Day 1 to Week 12 when defining responders using the ACR20. Sensitivity to change was also supported when using the DAS28-hsCRP as an anchor, except for comparisons between the lowest disease activity groups.

However, although these symptoms have been demonstrated to be important to patients with RA, an assessment of these symptoms was excluded from the the core set of disease activity measures. Specifically, rheumatologists have frequently used morning stiffness as an indicator that patient medication changes are needed [25]. However, in 1993, the core set of disease activity measures for use in RA clinical trials was revised. Studies with therapies available at the time (e.g., csDMARDs, auranofin, nonsteroidal anti-inflammatory drugs [NSAIDs]) suggested that MJS was not sensitive to change with treatment compared to control, and this lack of ability to discriminate treatment from control contributed to this symptom’s exclusion from the core set of measures [26].

Subsequent to its removal from the ACR core set in 1993, morning stiffness has been found to be an important determining clinical status indicator driving changes to DMARD therapy [27]. Additional work by Boers et al. [28] further demonstrated that both duration and severity of morning stiffness are related to other measures of disease activity. The findings from the present study provide support that both the Duration of MJS and Severity of MJS PROs are not only associated with other measures of disease activity in RA but rather are measures that assess distinct symptom experiences by patients. Furthermore, these measures are sensitive to detect change over time in adults with moderately to severely active RA. However, future research is needed in order to determine the relationship of these instruments to patient outcomes over a long term period, and the usability of such instruments in a clinical setting. Given the advent of electronic PRO diaries, these instruments could be used in a clinical setting and collected daily, thus enhancing the dialogue between patients and care providers. Further, using applications on mobile phones, these instruments can be easily completed by patients to track and collect their daily symptoms.

Despite the strong findings related to the reliability, validity, and responsiveness of the Duration of MJS and Severity of MJS PROs, a key limitation to this investigation is the missing data at the Day 1 assessment. As noted above, the missing data were due to a variety of reasons (e.g., device alarm not sounding until the following day). However, sensitivity analyses were conducted after imputing missing Day 1 Duration of MJS and Severity of MJS PRO scores, and results for the evaluations of reliability, validity, and responsiveness of these two PROs remained the same. In addition, the evaluation of responsiveness was limited as the studies’ baseline assessment period consisted of only data at Day 1 rather than a full 7 days of data as was available at Week 12. Finally, all analyses were pooled across countries due to small sample sizes (<5 participants) within certain participating countries. Thus, future research is needed to assess the psychometric properties of both instruments within each country.

## Conclusion

The findings from the current study demonstrate that the Duration of MJS and Severity of MJS items are unique PROs fit for purpose of measuring two key symptoms of MJS in adult patients with moderately to severely active RA in clinical trials.

## Additional file

Additional file 1: Table S1. Descriptive Statistics for Duration of MJS and Severity of MJS at Day 1. **Table S2** Reasons for Missing Diary Data at Day 1 for RA-BEAM and RA-BUILD. (DOCX 24 kb)

## Abbreviations

ACR: American College of Rheumatology
ACR20: 20% improvement in American College of Rheumatology criteria
ANCOVA: Analysis of covariance
ANOVA: Analysis of variance
CDAI: Clinical Disease Activity Index
csDMARD: Conventional synthetic DMARD
DAS28: Disease Activity Score modified to include the 28 diarthroidal joint count
DMARD: Disease-modifying antirheumatic drug
DSM-IV: American Psychiatric Association’s Diagnostic and Statistical Manual of Mental Disorders, 4th Edition
ESR: Erythrocyte sedimentation rate
HAQ-DI: Health Assessment Questionnaire-Disability Index
HRQOL: Health-related quality of life
hsCRP: High-sensitivity C-reactive protein
ICC: Intraclass correlation coefficient
IL-6: Interleukin-6
MCS: Mental Component Score
MJS: Morning Joint Stiffness
NSAID: Nonsteroidal anti-inflammatory drug
PCS: Physical Component Score
PhGA: Physician’s Global Assessment of Disease Activity
PRO: Patient-reported outcome
PtGA: Patient’s Global Assessment of Disease Activity
QIDS-SR_16_: Quick Inventory of Depressive Symptomatology Self-Rated-16
RA: Rheumatoid arthritis
SDAI: Simplified Disease Activity Index
SF-36: Medical Outcomes Study 36-Item Short Form Health Survey Version 2 Acute
SJC: Swollen joint count
TJC: Tender joint count
TNF: Tumor necrosis factor
VAS: Visual analogue scale
