# Supplementary material for: Psychometric properties of morning joint stiffness duration and severity measures in patients with moderately to severely active rheumatoid arthritis
Source: Health Qual Life Outcomes. 2017 Dec 6;15:239. doi: 10.1186/s12955-017-0813-7 (PMC5719576; doi:10.1186/s12955-017-0813-7)
Supplement: Additional file 1: Table S1. — Descriptive Statistics for Duration of MJS and Severity of MJS at Day 1. Table S2 Reasons for Missing Diary Data at Day 1 for RA-BEAM and RA-BUILD. (DOCX 24 kb) [file 12955_2017_813_MOESM1_ESM.docx]

Additional file 1

Table S1. Descriptive Statistics for Duration of MJS and Severity of MJS at Day 1

| Study | PRO | n | Mean (SD) | Median | Range | Floor (%)^a^ | Ceiling (%)^a^ |
| --- | --- | --- | --- | --- | --- | --- | --- |
| RA-BEAM | Duration of MJS | 537 | 152.8 (180.8) | 90.0 | 0-720 | 1.1% | 1.8% |
|  | Severity of MJS | 537 | 5.8 (2.2) | 6.0 | 0-10 | 0.8% | 1.5% |
| RA-BUILD | Duration of MJS | 311 | 160.7 (174.8) | 91.0 | 0-720 | 0.7% | 1.8% |
|  | Severity of MJS | 312 | 5.7 (2.1) | 6.0 | 0-10 | 0.3% | 1.3% |

Abbreviations: MJS = morning joint stiffness; n = number of patients in the specified category; SD = standard deviation.

^a^ For all scores, the floor effect was assessed based on the minimum score and the ceiling effect was assessed ased on the maximum score possible for the range.

Table S2. Reasons for Missing Diary Data at Day 1 for RA-BEAM and RA-BUILD

| Reason, n (%) | RA-BEAM N=768 | RA-BUILD N=372 |
| --- | --- | --- |
| Device never given to patient | 17 (2.2) | 12 (3.2) |
| Device given to patient after baseline (Day 1) | 130 (16.9) | 57 (15.3) |
| Missed alarms | 97 (12.6) | 48 (12.9) |
| Low battery | 82 (10.7) | 25 (6.7) |
| First alarm next day | 256 (33.3) | 208 (55.9) |
| User abandoned or delayed report past window | 171 (22.3) | 21 (5.6) |
| Other | 15 (2) | 1 (0.3) |
